# Clinical study on sequential treatment of severe diarrhea irritable bowel syndrome with precision probiotic strains transplantation capsules, fecal microbiota transplantation capsules and live combined bacillus subtilis and enterococcus faecium capsules

**DOI:** 10.3389/fcimb.2022.1025889

**Published:** 2022-09-28

**Authors:** Linling Lv, Guangcong Ruan, Yi Ping, Yi Cheng, Yuting Tian, Zhifeng Xiao, Xuefei Zhao, Dongfeng Chen, Yanling Wei

**Affiliations:** Department of Gastroenterology, Chongqing Key Laboratory of Digestive Malignancies, Daping Hospital, Army Medical University (Third Military Medical University), Chongqing, China

**Keywords:** Irritable bowel syndrome, precision transplantation, fecal microbiota transplantation capsules, live combined bacillus subtilis and enterococcus faecium capsules, gut microbiota

## Abstract

**Objective:**

To study the effect of precision probiotic strains transplantation capsules on diarrhea irritable bowel syndrome compared with fecal microbiota transplantation capsules and live combined bacillus subtilis and enterococcus faecium capsules.

**Methods:**

Two patients with severe irritable bowel syndrome were treated with precision probiotic strains transplantation capsules, fecal microbiota transplantation capsules and live combined bacillus subtilis and enterococcus faecium capsules in sequence. IBS-SSS, IBS-QoL, GSRS, stool frequency, stool character, degree of abdominal pain, GAD-7, and PHQ9 scores of patients at 0, 2, 4, 6, 8, 10, and 12 weeks of treatment were monitored and recorded, and stool samples were collected for metagenomics and metabolomics.

**Results:**

It was found that the IBS-SSS score of patient case 1 decreased by 175 points and that of patient case 2 decreased by 100 points after treatment of precision probiotic strains transplantation capsules. There was no significant decrease after fecal microbiota transplantation capsules and live combined bacillus subtilis and enterococcus faecium capsules were used. At the same time, compared with fecal microbiota transplantation and live combined bacillus subtilis and enterococcus faecium capsules, the IBS QoL, stool frequency, stool character, degree of abdominal pain and GAD-7 score of patient case 1 improved more significantly by the precision probiotic strains transplantation capsules. And the stool frequency and stool character score of patient case 2 decreased more significantly. Intestinal microbiota also improved more significantly after the precise capsule transplantation treatment. And we found *Eubacterium_ Eligens* showed the same change trend in the treatment of two patients, which may play a role in the treatment.

**Conclusion:**

precision probiotic strains transplantation capsules is more beneficial to improve the intestinal microbiota of patients than microbiota transplantation capsule and live combined bacillus subtilis and enterococcus faecium capsules, so as to better alleviate clinical symptoms. This study provides a more perfect and convenient therapeutic drugs for the treatment of IBS.

## Introduction

Irritable bowel syndrome (IBS) is a common functional gastrointestinal (GI) disorder characterized by recurrent abdominal pain associated with changes in stool frequency and form (Hellström et al., 2019). According to the predominant bowel habits, IBS is divided into diarrhea (IBS-D), constipation (IBS-C), mixed (IBS-M), and irregular (IBS-U).10%-15% of people worldwide are troubled by IBS (Defrees and Bailey, 2017). According to current research, the gut microbiota, gastrointestinal motility, visceral paresthesia, changes in intestinal permeability and brain-gut axis, and even infection or psychological stress may be related to the occurrence of IBS (Ford et al., 2017). The current management of IBS is include diet and lifestyle changes, probiotics, psychotherapy and medications (opioid receptor agonists, smooth muscle antispasmodics, bile acid chelators, antidepressants, 5-HT3 antagonists, etc.) (Cangemi and Lacy, 2019).

More and more studies suggest the influence of microbiota on IBS. The Rome Foundation Working Team acknowledged that the dysbiosis of microbiota is a possible contributor to IBS (Simrén et al., 2013). The ratio of *Firmicutes* and *Bacteroidetes* in the microbiota of IBS patients increased, and the ratios of *Clostridiales* and *Bifidobacterium* decreased (Rajilić-Stojanović et al., 2011). So treatments based on regulating the microbiota may be effective for IBS. Ruggiero et al. used a probiotic mixture of Bacillus subtilis and Streptococcus faecium to treat patients with IBS with celiac disease. Compared with placebo, the effective rate was significantly higher (15.3% vs. 3.8%; P<0.04) (Francavilla et al., 2019).

Although probiotics are effective in treatment, their effective rate is low. At present, an emerging treatment method fecal microbiota transplantation (FMT), is gradually applied in clinical practice. FMT is an established treatment for recurrent Clostridium difficile infection which cure rate reaches 80–90% (Quraishi et al., 2017). FMT was also be tried to use to treat IBS with an effective rate of 65% (Johnsen et al., 2018). By adjusting the FMT dose, the effective rate can be increased to 89.1% (El-Salhy et al., 2020).

Although the above studies have suggested the possible therapeutic effect of FMT on IBS, the current understanding of fecal bacteria used for FMT treatment can hardly provide precise quantitative data or quantitative indicators of colonization for this microbial population, and different donors are quite different. It is difficult for fecal bacteria to meet the standards in terms of preparation, standard type and quality control. Therefore, there is an urgent need to accurately supplement the gastrointestinal microbiota in response to the changes in the stool of IBS patients. Selective microbiota transplantation (SMT), that is, by supplementing IBS-friendly bacteria and quantifying them to patients, precise treatment of IBS can be achieved.

In this article, we describe two cases of IBS patients, and report the comparison of their efficacy after using our self-developed precision probiotic strains transplantation capsules, fecal microbiota transplant capsules, and live combined bacillus subtilis and enterococcus faecium capsules. We also described the changes in intestinal microbes and inflammation indicators before and after treatment.

## Material and methods

### Precision strains transplantation capsules

Based on most studies, the difference in the microbiota between IBS patients and normal people is mainly manifested in the decrease in the number of *Lactobacillus* and *Bifidobacterium*. We plan to supplement the microbiota of *Lactobacillus* and *Bifidobacterium*. Screen the *Bifidobacterium* and *Lactobacillus* species in the ‘‘The list of strains that can be used for food in China’’. Strains that are mainly used for diarrhea or have immunomodulatory effects were selected, and finally 9 kinds of probiotics were included: *Bifidobacterium breve, Bifidobacterium infantis, Bifidobacterium longum, Lactobacillus acidophilus, Lactobacillus casei, Lactobacillus helveticus, Lactobacillus plantarum, Lactobacillus reuteri and Lactobacillus rhamnosus*. Mix 9 kinds of strains powder to make precision strains transplantation capsules (IBS-diarrhea type). The main components of the precision strains transplantation capsules are: each capsule contains 45mg of each strain, each capsule contains 400mg of 9 strains, and the capsule shell is enteric-coated capsules. The study was conducted from November 2020 to March 2021 at Daping Hospital, Chongqing, China, and was approved by the Ethics Committee of Daping Hospital Affiliated Army Medical University (Third Military Medical University. All study participants provided written informed consent.

### Fecal microbiota transplantation donor

According to the FMT donor guidelines, a donor was recruited and screened. By exploring his medical history and living habits, any possibility of contact with infectious pathogens or dangerous social or sexual behaviors (such as drug abuse) can be ruled out. He also underwent physical examinations and blood tests to rule out gastrointestinal, metabolic or neurological diseases (complete blood count, blood sugar, electrolytes and inflammation markers). Serological screenings for HIV, syphilis and hepatitis A, B and C were also carried out. The results of all these tests and inspections are negative. The donor was a 22-year-old Chinese soldier. He was non-smoker, healthy, did not take any drugs, and had a body mass index of 21 kg/m^2^. He has not relationship with any of the patients in the trial. He trains five times a week for 1 hour each time. Regular diet and work and rest. The donor has donated his stool within 12 months, and his stool samples are tested every 3 months. The sample maintains a normal biomass, with only minor changes in the composition of bacteria.

### Live combined bacillus subtilis and enterococcus faecium capsules

Live combined bacillus subtilis and enterococcus faecium capsules are commonly used intestinal probiotics, containing bacillus subtilis R-179 (5.0×10^7 cfu) and enterococcus faecium R-026 (4.5×10^^8^ cfu), which can regulate the human intestinal tract The environment promotes the growth and reproduction of normal Gastrointestinal microbiota and inhibits the growth of intestinal pathogenic bacteria, thereby effectively protecting the intestinal tract.

### Therapeutic schedule

Two patients were given sequential treatment with three drugs. First, the precision probiotic strains transplantation capsules (diarrhea type) was given orally, once a day, 4 capsules each time, after 2 weeks of use, the drug was stopped for 2 weeks to elute the effect of the drug. Then in the fourth week, he was treated with fecal microbiota transplantation capsules once, and after two weeks of observation, he was given another two-week drug washout period. In the 8th week, the treatment with live combined bacillus subtilis and enterococcus faecium capsules was started, 3 times a day, 2 capsules each time, and after the same use for 2 weeks, the drug was stopped for 2 weeks. Monitor the changes in clinical symptoms of IBS before use, 2 weeks after use, and 2 weeks after drug withdrawal, and record the number of stools and changes in stool characteristics. Used IBS-SSS, GSRS, IBS-QoL, GAD7, PHQ9, SAS, and SDS scales monitor the changes in bowel symptoms before and after treatment, changes in life treatment, and changes in anxiety and depression. In addition, stool specimens were collected for microbiota analysis, and changes in inflammatory factors were monitored at the same time. Safety indicators (including liver function, kidney function, electrolytes, blood routine, etc.) are also monitored during drug use, and adverse reactions are recorded at any time.

### Clinical response criteria

The main research index is the severity of the patient’s disease, which is evaluated using the IBS-SSS score (Francis et al., 1997). Secondary indicators include the patient’s stool frequency, stool characteristics, and abdominal pain score. The Bristol Stool Trait Scale and Facial Expression Scale were used to evaluate fecal traits and abdominal pain. In addition, we used the IBS-QoL scale to evaluate their quality of life, and used the GAD-7 and PHQ-9 scales to evaluate the changes in their anxiety and depression status.

The patient’s IBS-SSS score decreased by at least 50 points or the severity decreased by one level, indicating clinical remission. The times of stools more than 4 drops to 4 or less, the abdominal pain score is dropped by 30%, GSRS score is dropped by 30%, and the stool characteristics are changed to types 3, 4, and 5 for patients which with stool types 6 and 7 were consider effective. A 30% drop in IBS-QoL score indicates an improvement in quality of life. The improvement of anxiety and depression is manifested in the degree of anxiety and depression drops at least one level.

### Metagenomic and metabolomic analysis

The fecal samples of patients were analyzed by metagenomics and metabolomics. Select small fragment library on SL5 platform for sequencing (Nuohe Zhiyuan biology company, China). Basic quality control, species and function notes: use fastp (version 0.20.0) to control the original data, and the parameter is cut_ tail -W4 -M20 -n5 -c -l50 -w6. Bmtagger (version 1.1.0) is used to remove the host from the data after quality control, and the parameter is default. The human genome (hg19) metaphlan (version 3.0) was used for species annotation. Humann (V3.0.0. Alpha. 4) for function annotation. The metabolic data acquisition instrument system mainly includes ultra-performance liquid chromatography (UPLC) and tandem mass spectrometry (MS/MS). Based on the self-built targeted target database MWDB (metware database), the information and secondary spectrum data are analyzed qualitatively according to the retention time RT (retention time) and parent and daughter ions of the detected substances.

### Statistical analysis

R version 4.0.2 was used for correlation analysis and mapping. Alpha diversity calculation: package vegan version 2.5-7, based on the species level abundance table. Bray Curtis distance calculation: package vegan version 2.5-7, based on the species level abundance table. PCoA analysis: package ape version 5.5. Heat map drawing: package pheatmap version 1.0.12. Other drawings: package ggplot2 version 3.3.5. Spearman statistical method was used to calculate the correlation between metabolites and total abundance of different flora. P < 0.05, suggesting that the difference was statistically significant.

## Results

### Case presentation

Case 1: A 31-years-old male patient. The patient had abdominal pain and diarrhea starting from 20 months before admission without obvious triggers. The abdominal pain was a dull pain around the umbilical cord, which could be relieved after defecation. The diarrhea continued to occur, up to 10+ times/day. It was loose or watery stools, occasionally adheres mucus or foam, with no pus or blood, no tenesmus, no fever, nausea, or vomiting, etc. Gastrointestinal endoscopy and abdominal CT showed no obvious abnormalities. He has been treated with probiotics, pinaverium bromide and other drugs, with poor efficacy. Only three sequential drugs were used during this study.

Case 2: A 20-years-old male patient. Patients have recurrent diarrhea for 1+ years, mostly after a cold, spicy diet, with intermittent attacks, about 4-7 times/day, and 9 times/day at most; stools are mostly mushy stools with mucus; with abdominal pain and bloating, and they are slightly relieved after defecation. Gastrointestinal endoscopy and abdominal CT showed no obvious abnormalities. Probiotics, pinaverium bromide and other drugs have also been used. Only three sequential drugs were used during this study.

### Main indicators

**Figure 1:**
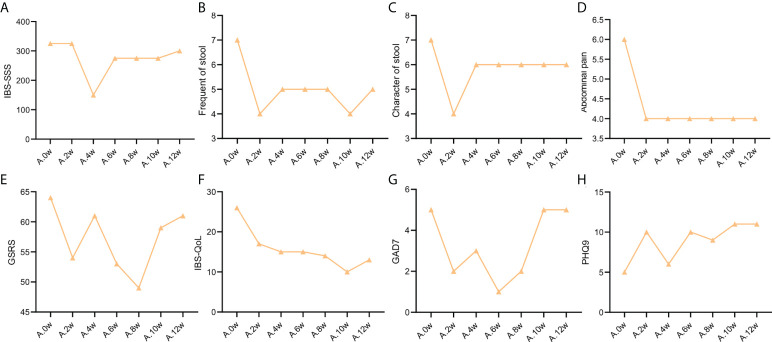
Change trend of IBS clinical indicators of patient case 1. **(A)**. IBS-SSS score. **(B)**. stool frequency. **(C)**. Character of stool. **(D)**. Abdominal pain score. **(E)**. GSRS. **(F)**. IBS-QoL. **(G)**. GAD7. **(H)**. PHQ9.

**Figure 2:**
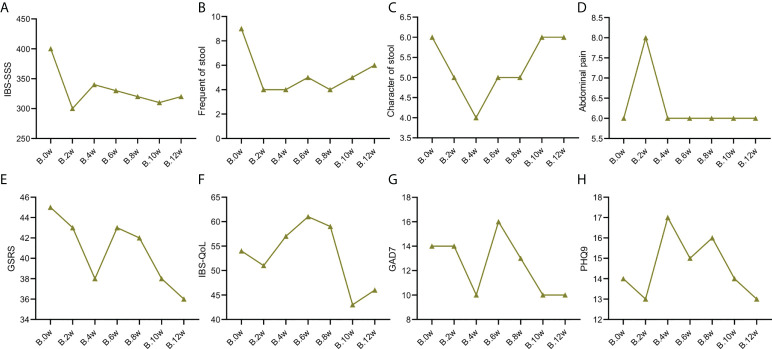
Change trend of IBS clinical indicators of patient case 2. **(A)**. IBS-SSS score. **(B)**. stool frequency. **(C)**. Character of stool. **(D)**. Abdominal pain score. **(E)**. GSRS. **(F)**. IBS-QoL. **(G)**. GAD7. **(H)**. PHQ9.

The severity of the disease of the two patients was significantly improved after using the precision probiotic strains transplantation capsules, which was not obvious after standard fecal microbiota transplantation and live combined bacillus subtilis and enterococcus faecium capsules. As shown in **Table S1**, the IBS-SSS score of case 1 patient dropped by 175 points, achieving remission after the use of precision probiotic strains transplantation capsules; but there was no remission after standard fecal microbiota transplantation and live combined bacillus subtilis and enterococcus faecium capsules. The case 2 patient also achieved remission (IBS-SSS score dropped by 100 points) after treated with precision probiotic strains transplantation capsules. Even more, after stopping the drug for 2 weeks, there was still a score reduction of 60 points. In comparison, there were only 10 points reduction after standard fecal microbiota transplantation and live combined bacillus subtilis and enterococcus faecium capsules. The IBS-SSS score trend of the two patients before and after the 3 treatments is shown in **Figure 1A** and **Figure 2A**.

### Secondary indicators

As shown in **Table S2**, the frequency of stools in the two patients was reduced to 4 times/day after treatment with precision probiotic strains transplantation capsules. Fecal microbiota transplantation is still effective for the case 2 patient, that it can maintain the stool frequency at 4 times/day. But it was increase to 5 times/day after the case 1 patient used fecal microbiota transplantation. Both of them got not remission after live combined bacillus subtilis and enterococcus faecium capsules treatment. The change trend of stool frequency is shown in **Figure 1B** and **Figure 2B**.

For the case 1 patient, although the frequency of defecation decreased significantly, but after the use of the three drugs, the stool properties were not significantly improved, only from watery stool to mushy stool (**Table S2**, **Figure 1C**). In contrast, the times of stools of the case 2 patient decreased, and the stool character also improved significantly. After the use of precision probiotic strains transplantation capsules, the patient changed from pasty stool to visible broken edge massive stool (**Table S2**, **Figure 2C**).

Both of the two patients had obvious abdominal pain before treatment. Both of them got 6 of the Facial Expression Scale. It dropped to 4 after the use of the precision transplant capsule in the case 1 patient (**Table S2**, **Figure 1D**). But there was no significant change after the use of the latter two drugs. The case 2 patient had no relief of abdominal pain after three treatments (**Table S2**, **Figure 2D**).

GSRS scores of both patients showed no significant improvement in gastrointestinal symptoms (Figure **Figures 1E**, **2E**)

Irritable bowel syndrome also has a significant impact on the quality of life of patients. **Table S3** shows that for the case 1 patient, the IBS QoL score decreased after the use of precision probiotic strains transplantation capsules, suggesting that the quality of life improved significantly, 34.6% after 2 weeks and 42.3% after 4 weeks. However, there was no significant improvement after the other two treatments. The case 2 patient had no improvement in quality of life after three treatments. The change trend of stool frequency is shown in **Figure 1F** and **Figure 2F**.

The standard for evaluation of anxiety and depression of GAD-7 and PHQ-9 was 0-4 normal, 5-9 mild, 10-14 moderate, 15-27 severe. Case 1 patient suffered a mild anxiety and depression. The anxiety was obviously relieved after the use of precision probiotic strains transplantation capsules, that the other two treatments were ineffective (**Table S4**, **Figures 1G**, **H**). The case 2 patient suffered a moderate anxiety and depression. The precision probiotic strains transplantation capsules and standard fecal microbiota transplantation treatment had no effect on his anxiety and depression, but live combined bacillus subtilis and enterococcus faecium capsules improved the patient’s depression (**Table S4**, **Figures 2G**, **H**).

### Gut microbiota

In order to evaluate the changes of gut bacterial community after different treatment. We collected stool samples from patients at each follow-up time point for macrogenomic sequencing analysis.

**Figure 3:**
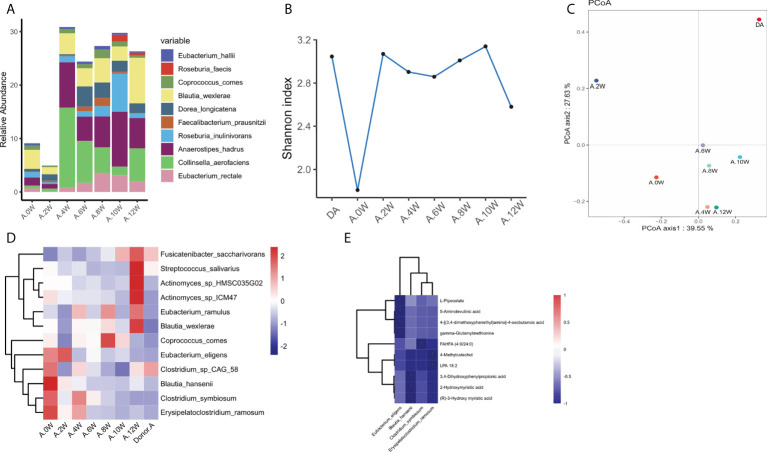
Characteristics of gastrointestinal microbiota in patient case 1. **(A)**. Composition of the top 10 species at the species level. **(B)**. The diversity (Shannon index) of gastrointestinal microbiota. **(C)**. Principal coordinate analysis (PCoA) was used to investigate the similarity of microbial communities at different time points. **(D)**. Strains with the same change trend before and after treatment with three treatments. **(E)**. Correlation between strains with the same change trend and metabolites in neg mode. *p < 0.05.

**Figure 4:**
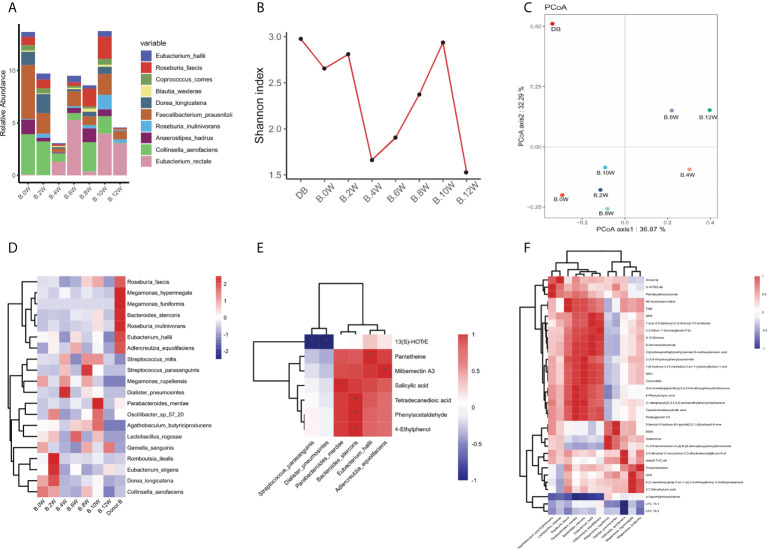
Characteristics of gastrointestinal microbiota in patient case 2. **(A)**. Composition of the top 10 species at the species level. **(B)**. The diversity (Shannon index) of gastrointestinal microbiota. **(C)**. Principal coordinate analysis (PCoA) was used to investigate the similarity of microbial communities at different time points. **(D)**. Strains with the same change trend before and after treatment with three treatments. **(E)**. Correlation between strains with the same change trend and metabolites in pos mode. **(F)**. Correlation between strains with the same change trend and metabolites in neg mode. *p < 0.05.

The composition of gastrointestinal microbiota was different before and after treatment. At the phylum level, we can observe that the gastrointestinal microbiota of the two patients was mainly composed of *Firmicutes*, *Bacteroides*, *actinomycetes* and *Proteus.* After treatment, it was found that the abundance of *Bacteroidetes* in patient case 1 decreased after the use of precision probiotic strains transplantation capsules, but there was no such change after FMT and live combined bacillus subtilis and enterococcus faecium capsules treatment (**Figure S1A**). In patient case 2, it was observed that the abundance of *Firmicutes* decreased after the use of precision probiotic strains transplantation capsules, but increased after the other two treatments (**Figure S1C**). The picture shows the changes of major microbiota at the genus level of gastrointestinal microbiota in two patients. Before treatment, patient case 1 was mainly composed of *Blautia* and *Bacteroides*. *Blautia* decreased after treatment with live combined bacillus subtilis and enterococcus faecium capsules, while *Bacteroides* decreased after treatment with precision probiotic strains transplantation capsules (**Figure S1B**). However, in patient case 2, *Blautia* decreased after precision capsule transplantation (**Figure S1D**). At the level of species, we listed the main strains contained in the intestine of patient a and found that *Blautia_wexlerae* was the most abundant strain. It decreased after treatment with precision probiotic strains transplantation capsules, but this change was not found after the other two treatments (**Figure 3A**). In patient B, the abundance of *Faecalibaterium prausnitzii* was higher, but decreased at four weeks, eight weeks and twelve weeks, that is, after the withdrawal of each treatment (**Figure 4A**).

In patient case 1, the diversity (Shannon index) of gastrointestinal microbiota increased most significantly after precision probiotic strains transplantation capsule, and decreased after drug withdrawal (**Figure 3B**). The diversity of gastrointestinal microbiota also increased after the treatment of patient case 2 precision probiotic strains transplantation capsules (**Figure 4B**). Principal coordinate analysis (PCoA) was used to investigate the similarity of microbial communities at different time points. No obvious time point aggregation was found, but there was obvious heterogeneity before and after treatment (**Figures 3C**, **4C**).

We analyzed the changes of gastrointestinal microbiota species before and after each treatment to further evaluate the colonies that may play a role in the treatment. The **Figure S2** and **Figure S3** show the differences of strains at the level of phylum, and the top 20 microbiota with the most obvious differences at the level of genus and species. In patient case 1, 12 strains with the same change trend before and after treatment were screened (**Figure 3D**). *Fusicatenibacter_ saccharivorans Eubacterium_ eligens Actinomyces_ sp_ Hmsc035g02* were up-regulated after treatment, while *Lostridium_ sp_ CAG_ 58, Clostridium_ symbiosum, Blautia_ hansenii, Coprococcus_ comes, Eubacterium_ ramulus, Erysipelatoclostridium_ ramosum, Blautia_ wexlerae, Streptococcus_ salivarius, Actinomyces_ sp_ Icm47* were down regulated after treatment. The correlation between related strains and bacterial metabolites was analyzed. Four strains *Eubacterium-eligens, Blautia-hanserii, Clostridium-symbiosum* and *Erysipelatoclostridium* were found to be negatively correlated with metabolites (**Figure 3E**). In patient case 2, 20 strains with the same change trend before and after treatment were selected (**Figure 4D**). *Eubacterium_ hallii, Agathobaculum_ butyriciproducens, Arabacteroides_ merdae, Bacteroides_ stercoris, Roseburia_ inulinivorans, Dorea_ longicatena, Romboutsia_ ilealis, Oscillibacter_ sp_ 57_ 20, Adlercreutzia_ equolifaciens, Lactobacillus_ rogosae, Eubacterium_ eligens, Roseburia* were up-regulated after treatment, while *Megamonas_ hypermegale, Megamonas_ funiformis, Streptococcus_ mitis, Streptococcus_ parasanguinis, Gemella_ sanguinis, Megamonas_ rupellensis, Collinsella_ aerofaciens, Dialister_ Pneumosintes* were down regulated after treatment. The picture shows their correlation with metabolites. Among them, 6 strains are related to metabolites in pos mode (**Figure 4E**). *Streptoccus_ Parasanguinis* and *dialist_ Pneumosintes* decreased after treatment and were negatively correlated with 13 (S)-HOTrE. *Parabacteroides _ merdae, Bacteroides_ stercoris, Eubacterium_ hallii, Adlercreutzia_ Equolifaciens* were positively correlated with the corresponding metabolites. 12 strains are related to metabolites in neg mode (**Figure 4F**).

**Figure 5:**
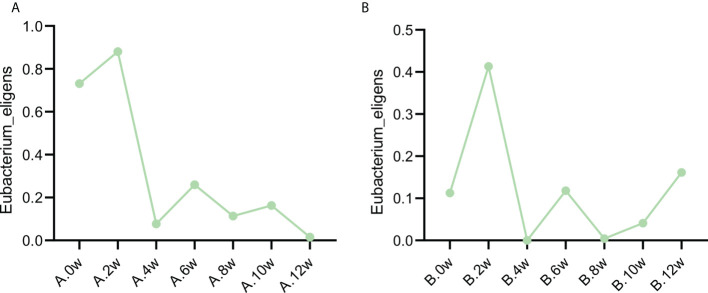
Variation trend of Eubacterium eligens before and after different treatment. **(A)** Case 1, **(B)** Case 2.

Interestingly, it is found that the same change trend of *Eubacterium_eligens* before and after different drug treatments in the two patients. **Figure 5** shows the change at each time point.

### Side reaction

The two patients had no obvious side effects during the study.

## Discussion

IBS is a chronic intestinal functional disease (Mearin et al., 2016; Holtmann et al., 2016). The incidence rate of the world is high, and its impact on quality of life is comparable to other chronic diseases, such as diabetes and liver diseases. The economic burden is also very heavy (Everhart and Ruhl, 2009). IBS is characterized by abdominal pain, abdominal distension, abdominal discomfort and irregular stool. The two patients we observed were mainly IBS-D patients with abdominal pain and diarrhea. According to their IBS-SSS score, they were severe IBS-D patients. According to the brief GAD-7 and PHQ-9 scores, patient case 1 was complicated with mild anxiety and depression, while patient case 2 was complicated with moderate anxiety and depression. Although the pathophysiology of irritable bowel syndrome has not been fully clarified, researchers increasingly believe that the imbalance of gastrointestinal microbiota is related to it.

Gastrointestinal microbiota is a diverse and numerous ecosystem, which is distributed in the whole gastrointestinal tract and has an impact on all systems of our body. However, due to its huge complexity and high variability among individuals, the role of gastrointestinal microbiota in human physiology has not been fully understood. In this experiment, the use of three drugs was designed to regulate the gastrointestinal microbiota, observe their curative effects, and detect the changes of microbiota before and after use to further clarify the role of microbiota.

Gut microbiota refers to bacteria, viruses, parasites (Sender et al., 2016). The adult gut microbiota is composed of more than 2000 kinds of bacteria, with increasing density and diversity from the stomach to the colon. Healthy gastrointestinal microbiota is a microbial ecosystem with diversity, stability, resistance and adaptability (Lozupone et al., 2012). Gastrointestinal microbiota in abiotic state is increasingly involved in the pathogenesis and development of many diseases. Animal model experiments show that transplanting the gastrointestinal microbiota of IBS patients into sterile animals can cause visceral hypersensitivity, damage intestinal permeability and change gastrointestinal transport time, indicating the importance and possible etiological role of gastrointestinal microbiota in IBS (Crouzet et al., 2013). The microbiota characteristics of IBS patients are as follows: *Clostridium, Bacteroides, Bifidobacterium* and *Faecalibacterium* are significantly reduced in IBS (Rajilić-Stojanović et al., 2011; Jalanka-Tuovinen et al., 2014), while the bacteria associated with *Ruminococcus torques* (a species of *Lachnospiraceae*) are abundant in IBS patients (Scully et al., 2010; Rajilić-Stojanović et al., 2011; Saulnier et al., 2011), and the level is positively correlated with intestinal symptoms (Malinen et al., 2010; Rajilić-Stojanović et al., 2011; Saulnier et al., 2011). In addition, the study found that an increase in the ratio of *Firmicutes* to *Bacteroidetes* has been observed at the phylum level (Rajilić-Stojanović et al., 2011). Therefore, the Rome Foundation Working Team (Simrén et al., 2013) also believes that microbiota imbalance is a reasonable pathogenic factor of IBS.

The use of probiotics can improve the symptoms of IBS patients. Probiotic products containing lactic acid bacteria can significantly reduce specific symptoms (i.e. abdominal pain and flatulence) and improve the quality of life of patients (Asha and Khalil, 2020). *Lactobacillus* is reported to be beneficial to abdominal pain in functional gastrointestinal diseases (Horvath et al., 2011; Jadrešin et al., 2017). Probiotics containing *Lactobacillus* or *Bifidobacterium* were found to increase stool frequency and reduce intestinal transit time (Miller et al., 2017).

FMT is the transfer of fecal microbial content from healthy donors to individuals with gastrointestinal diseases. The mechanism of action is not completely clear, but the recovery of disordered microbiota seems to be the basis of the observed effect (Quraishi et al., 2017). FMT is mainly used to treat patients with recurrent *Clostridium difficile* infection (rCDI) (Debast et al., 2014; McDonald et al., 2018), and the cure rate is 80 - 90% (Quraishi et al., 2017). FMT can be taken orally through duodenal tube or capsule through the upper digestive tract (Kao et al., 2017) (van Nood et al., 2013). A study has shown that patients treated with oral capsule FMT have a high cure rate, and this method may reduce patients’ discomfort (Kao et al., 2017).Our study also used oral fecal bacteria capsule to treat patients with FMT. Johnsen et al. Published the first RCT to study the effect of FMT in patients with IBS (Johnsen et al., 2018). The authors found an overall improvement in symptoms in 58% of patients treated with FMT. A meta-analysis of five clinical studies found that fresh or frozen donor feces delivered through colonoscopy or naso jejunal bowel may be beneficial to IBS (Ianiro et al., 2019). Although FMT seems safe and easy to implement, it should be used with caution because its long-term effects are unclear or unidentifiable. FMT is not a standardized treatment, and the treatment methods are different in different places. The fecal bacteria used for transplantation are difficult to meet the standards in preparation, standard type and quality control. Using all the fecal bacteria of donors cannot provide accurate quantitative data or quantitative indicators of colonization, nor can it avoid harmful microorganisms, and the samples of different donors are quite different. That makes the application of FMT have some limitations.

We hope to design a selective microbiota transplantation capsule for IBS-D, so we designed accurate transplantation capsules according to the microbiota characteristics of IBS-D. We mainly screened *Bifidobacteria* and *Lactobacillus* which decreased in patients with IBS. Nine probiotics were screened, including: *Bifidobacterium breve, Bifidobacterium infantis, Bifidobacterium longum, Lactobacillus acidophilus, Lactobacillus casei, Lactobacillus helveticus, Lactobacillus plantarum, Lactobacillus reuteri, Lactobacillus rhamnosus*. Fuqiang yuan et al. Included five randomized controlled trials of treating IBS with probiotics containing *Bifidobacterium infantis* for meta-analysis. The results showed that compound probiotics containing *Bifidobacterium infantis* could effectively treat IBS without obvious adverse reactions (Yuan et al., 2017). *Lactobacillus helveticus* and *Lactobacillus rhamnosus* supplements can significantly reduce the duration of antibiotic related diarrhea like defecation (Ren et al., 2018). *Bifidobacterium longum* can significantly improve the depression and quality of life of patients with IBS-D (Pinto-Sanchez et al., 2017). *Lactobacillus acidophilus, Lactobacillus casei, Lactobacillus rhamnosus (* Preston et al., 2018) and *Lactobacillus plantarum (* Ducrotté et al., 2012) can improve intestinal symptoms and quality of life in patients with IBS. There is clear evidence that *Bifidobacterium breve* has strong immunomodulatory properties. The research of Jianjun Ren et al. Shows that *Bifidobacterium breve* mainly restores the Th1/Th2 balance by inhibiting Th2 response (Ren et al., 2018). *Bifidobacterium infantis* can reduce the ability of systemic pro-inflammatory biomarkers and make INF-α decreased in chronic fatigue syndrome and psoriasis, and IL-6 decreased in ulcerative colitis and chronic fatigue syndrome (Groeger et al., 2013). It is reported that *Lactobacillus reuteri* can prevent oxidative stress and inhibit the increase of intestinal oxidation products (Amaretti et al., 2013). *Lactobacillus helveticus* can control the intestinal microbiota and enhance the infant’s immune system (Xiao et al., 2017), and inhibit immune cell proliferation and proinflammatory cytokines (IL-6 and IL-1β) (Yamashita et al., 2014). *Bifidobacterium longum* can regulate the proliferation of mouse immune B cells and inhibit Th1 (IL-2, IFN- γ)/Th2 (IL-4, IL-10) cytokine imbalance and immune cytokine production (Choi et al., 2019). In conclusion, the probiotics we screened mainly have the role of treating diarrhea and regulating immunity. Through practice, it is found that the therapeutic effect is gratifying.

Based on the severity of IBS-SSS, it can be seen that both the two patients belong to patients with severe IBS. The IBS-SSS score decreased by 50 points was defined as clinical response (El-Salhy et al., 2020). Both the two patients had clinical response after the use of precision probiotic strains transplantation capsules, and the severity of their condition decreased. However, no obvious clinical reactions were found after the use of fecal microbiota transplantation capsule and live combined bacillus subtilis and enterococcus faecium capsules. At the same time, the patient’s stool characteristics, stool frequency and abdominal pain were also significantly improved after the use of precision probiotic strains transplantation capsules. Fecal microbiota transplantation capsule and live combined bacillus subtilis and enterococcus faecium capsules also improved the stool characteristics of patients. The improvement of quality of life was assessed by IBS-QoL scale. It was found that only patient case one’s quality of life improved after using precision probiotic strains transplantation capsules. In terms of the impact on patients’ anxiety and depression, we found that precise transplantation of capsule improved the anxiety and depression of the first patient, but for the second patient, live combined bacillus subtilis and enterococcus faecium capsules showed the effect of improving emotional disorders. No obvious effect was found in the use of fecal microbiota transplantation capsule. In general, precision capsule can improve the severity of IBS-D patients, reduce the number of stools, improve the characteristics of stools, relieve abdominal pain, improve the quality of life of patients, and improve the anxiety and depression of patients. Fecal microbiota transplantation capsule can improve the characteristics of stool, and live combined bacillus subtilis and enterococcus faecium capsules can play a certain role in improving anxiety and depression. This suggests that the effectiveness of precision probiotic strains transplantation capsules for IBS-D patients is better than fecal microbiota transplantation capsule and live combined bacillus subtilis and enterococcus faecium capsules. It has the advantages of more convenient production, controllable safety and more advantages in future application.

Based on the mechanism of regulating intestinal microbiota, we chose these three drugs for treatment, so we monitored the changes of intestinal microbiota and metabolites during treatment through macrogenomic analysis. Through the analysis of microbiota, it was found that there were great differences in intestinal microbiota after the three treatments. It has been found that the proportion of *Firmicutes* and *Bacteroidetes* increased significantly in the intestinal microbiota of IBS patients (Rajilić-Stojanović et al., 2011). In our trial, it was found that the *Bacteroidetes* level of patient case 1 decreased after the use of precision probiotic strains transplantation capsules, and the *Firmicutes* also decreased in the 4th week after treatment. This change was not found after treatment with the other two drugs. This suggests that the treatment of precision probiotic strains transplantation capsules seems to be changing the disordered microbiota structure of patients. In patient case 2, it was also found that the *Firmicutes* decreased significantly after the use of precision probiotic strains transplantation capsules. *Blautia* was found to be more abundant in the fecal microbiota of patients with irritable bowel syndrome and ulcerative colitis than in healthy individuals (Rajilić-Stojanović et al., 2011; Nishino et al., 2018). The intestinal microbiota of our two patients was dominated by *Blautia* at the genus level. *Blautia* decreased in patient case 1 after treatment with live combined bacillus subtilis and enterococcus faecium capsules, and decreased in patient case 2 after treatment with precision probiotic strains transplantation capsules. This suggests that precise transplantation of capsules promotes the reduction of adverse microbiota in patients. This change is also reflected in the level of species. In patient case 1, *Blautia_wexlerae* decreased after the use of precision probiotic strains transplantation capsules. After precise capsule transplantation, the microbiota structure of patients changed significantly, but the richness and diversity of microbiota increased.

Different treatments have different effects. In order to explore the functional microbiota, we analyzed the characteristics of different microbiota before and after treatment. Some strains have the same change trend before and after the use of the three treatment methods. Among patients case 1, 3 strains were up-regulated after treatment, while 9 strains were down regulated after treatment. Among patients case 2, 12 strains were up-regulated after treatment, while 8 strains were down regulated after treatment.

Further evaluating the relationship between intestinal microbiota and its metabolites, we found that some strains had a certain correlation with metabolites. In patient case 1, we found that *Eubacterium_ eligens, Blautia_ hansenii, Clostridium_ symbiosum, Erysipelatoclostridium_ Ramosum* was negatively correlated with metabolites. *Clostridium_ Symbiosum* decreased significantly after treatment, and its negatively related FAHFA (Fatty acid esters of hydroxy fatty acids) has anti-inflammatory effect and can inhibit the production of LPS induced pro-inflammatory cytokines in macrophages and dendritic cells (Yore et al., 2014). *Erysipelatoclostridium_ Ramosum* that reduced after treatment was negatively correlated with LAP (plasma lysophosphatidic acid) and 4_ Methylcatechol, both of which could promote tumor cell apoptosis (Zheng et al., 2004; Karatug Kacar et al., 2018). In patient case 2, *Streptoccus_ Parasanguinis* and *dialist_ Pneumosintes* decreased after treatment and were negatively correlated with 13(S)-HOTrE which can produce anti-inflammatory effect by inactivating NLRP3 inflammatory body complex (Kumar et al., 2016). Several strains increased after treatment, *Parabacteroides _ merdae, Bacteroides_ stercoris, Eubacterium_ hallii, Adlercreutzia_ Equolifaciens* were positively correlated with the corresponding metabolites. Milbemectin A3 has antifungal activity (Silva et al., 2013). Salicylic acid mainly destroys eicosanoic acid metabolism, thus changing the levels of prostaglandins and leukotrienes (Mitchell et al., 1993). Phenylacetaldehyde can enhance the postsynaptic effect of dopamine and act as a neuromodulator of catecholamine neurotransmission in the brain (Paterson et al., 1990). Many different strains showed significant positive correlation with metabolites, such as *parabacteroides_Merdae* increased after treatment, which was positively correlated with 4−phenylbutyric acid and taurochenodeoxycholic acid. 4−phenylbutyric acid can improve lipotoxicity and stimulate fatty acids β (He and Moreau, 2019). Taurochenodeoxycholic acid can inhibit proinflammatory cytokines (IL-1 β、 IL-6 and TNF- α) to inhibit inflammation (Liu et al., 2011). The change of different strains leads to the production of different metabolites, which affects the intestinal function.

In the analysis, we found that the change trend of *Eubacterium_eligens* was the same in the two patients, and increased after the treatment of three drugs. It shows that it may play an important role in the improvement of symptoms and microbiota after treatment. *Eubacterium_Eligens* were negatively correlated with 5 − aminolevulinic acid, which induced the accumulation of protoporphyrin IX in malignant tissues (Fratz et al., 2014). It has been found that *Eubacterium_eligens* is a professional pectin degrading agent, which may provide anti-inflammatory activity by promoting the production of IL-10 by epithelial cells (Chung et al., 2017). Therefore, after treatment, the increase of *Eubacterium_eligens* improves the anti-inflammatory activity of microbiota. Whether Eubacterium plays a certain role in IBS needs further experiments.

Since it is not easy to include patients in sequential therapy, only 2 patients were included in this study for observation. Fen Zhang et al. Also found the changes of intestinal flora after FMT treatment during the treatment of only one patient, providing a new idea for the diagnosis and treatment of severe colitis associated with graft-versus-host disease in the future (Zhang et al., 2021). Our research shows that, compared with fecal microbiota transplantation capsule and live combined bacillus subtilis and enterococcus faecium capsules, precision probiotic strains transplantation capsules can significantly improve the severity of IBS-D patients, reduce the number of stools, change the nature of stools and reduce abdominal pain. Moreover, precision probiotic strains transplantation capsules can improve the quality of life of patients, and may also have a certain effect on patients with anxiety and depression. On the other hand, it was found that precision probiotic strains transplantation capsules had more beneficial improvement on the intestinal micriobiota of patients than microbiota transplantation capsule and live combined bacillus subtilis and enterococcus faecium capsules, resulting in better clinical symptom relief. Compared with live combined bacillus subtilis and enterococcus faecium capsules, precision probiotic strains transplantation capsules has more abundant microbiota. Compared with fecal microbiota transplantation capsules, it is more convenient to prepare and can accurately quantify the dose. This study provides a more perfect and convenient treatment scheme for the treatment of IBS-D. However, there are still deficiencies in this study. The interval between discontinuation of the three drugs is short, and the delayed effect of the drugs has an impact on the treatment. The interval needs to be extended in future experiments. And a larger sample size is needed in the future to further verify the effect of precision probiotic strains transplantation capsules.

## Data availability statement

The datasets presented in this study can be found in online repositories. The names of the repository/repositories and accession number(s) can be found in the article/**Supplementary Material**.

## Ethics statement

The studies involving human participants were reviewed and approved by the Ethics Committee at the Army Medical University (Third Military Medical University) affiliated with Daping Hospital. In addition, the study was registered at the Chinese Clinical Trial Registry (www.chictr.org.cn); the trial registration number was ChiCTR2100043160, through which the trial protocol can be accessed. The patients/participants provided their written informed consent to participate in this study.

## Author contributions

Conceptualization: YW, DC, LL, and GR. Formal analysis: LL, GR, and XZ. Data curation: YT, ZX, LL, and GR. Writing—original draft preparation: LL and GR. Writing—review and editing: YW, DC, LL, GR, and YP. Supervision: YW and DC. Validation: YP, YC, YT, and ZX. Funding: YW. All authors contributed to the article and approved the submitted version.

## Funding

This study was supported by the Chongqing Science and Health Joint Project (2019ZDXM026), the Clinical Technology Innovation Cultivation Program of Army Medical University (Third Military Medical University) (CX2019JS222), and Foundation Strengthening Program Technology Field Fund Project (2021-JCJQ-JJ-1083).

## Conflict of interest

The authors declare that the research was conducted in the absence of any commercial or financial relationships that could be construed as a potential conflict of interest.

## Publisher’s note

All claims expressed in this article are solely those of the authors and do not necessarily represent those of their affiliated organizations, or those of the publisher, the editors and the reviewers. Any product that may be evaluated in this article, or claim that may be made by its manufacturer, is not guaranteed or endorsed by the publisher.

## Supplementary material

Supplementary Figure 1 Composition of the top 10 species at the phylum and genus level. **(A)**. Phylum level in patient case 1. **(B)**. Genus level in patient case 1. **(C)**. Phylum level in patient case 2. **(B)**. Genus level in patient case 2.

Supplementary Figure 2 The differences of strains at the level of phylum, and the top 20 microbiota with the most obvious differences at the level of genus and species in patient case 1. A1. The differences of strains at the level of phylum before and after use of precision probiotic strains transplantation capsule. A2. The top 20 microbiota with the most obvious differences at the level of genus before and after use of precision probiotic strains transplantation capsule. A3. The top 20 microbiota with the most obvious differences at the level of species before and after use of precision probiotic strains transplantation capsule. B1. The differences of strains at the level before and after use of fecal microbiota transplantation. B2. The top 20 microbiota with the most obvious differences at the level of genus before and after use of fecal microbiota transplantation. B3. The top 20 microbiota with the most obvious differences at the level of species before and after use of fecal microbiota transplantation. C1. The differences of strains at the level of phylum before and after use of live combined bacillus subtilis and enterococcus faecium capsules. C2. The top 20 microbiota with the most obvious differences at the level of genus before and after use of live combined bacillus subtilis and enterococcus faecium capsules. C3. The top 20 microbiota with the most obvious differences at the level of species before and after use of live combined bacillus subtilis and enterococcus faecium capsules.

Supplementary Figure 3 The differences of strains at the level of phylum, and the top 20 microbiota with the most obvious differences at the level of genus and species in patient case 2. A1. The differences of strains at the level of phylum before and after use of precision probiotic strains transplantation capsule. A2. The top 20 microbiota with the most obvious differences at the level of genus before and after use of precision probiotic strains transplantation capsule. A3. The top 20 microbiota with the most obvious differences at the level of species before and after use of prcesion probiotic strains transplantation capsules. B1. The differences of strains at the level before and after use of fecal microbiota transplantation. B2. The top 20 microbiota with the most obvious differences at the level of genus before and after use of fecal microbiota transplantation. B3. The top 20 microbiota with the most obvious differences at the level of species before and after use of fecal microbiota transplantation. C1. The differences of strains at the level of phylum before and after use of live combined bacillus subtilis and enterococcus faecium capsules. C2. The top 20 microbiota with the most obvious differences at the level of genus before and after use of live combined bacillus subtilis and enterococcus faecium capsules. C3. The top 20 microbiota with the most obvious differences at the level of species before and after use of live combined bacillus subtilis and enterococcus faecium capsules.

Supplementary Table 1 IBS-SSS score change.

Supplementary Table 2 Changes of IBS related symptoms.

Supplementary Table 3 Changes in quality of life scale scores IBS-QoL.

Supplementary Table 4 Changes in anxiety and depression scale scores.
