# Supplementary figures and images for: Clinical study on sequential treatment of severe diarrhea irritable bowel syndrome with precision probiotic strains transplantation capsules, fecal microbiota transplantation capsules and live combined bacillus subtilis and enterococcus faecium capsules
Source: Front Cell Infect Microbiol. 2022 Sep 28;12:1025889. doi: 10.3389/fcimb.2022.1025889 (PMC9555570; doi:10.3389/fcimb.2022.1025889)

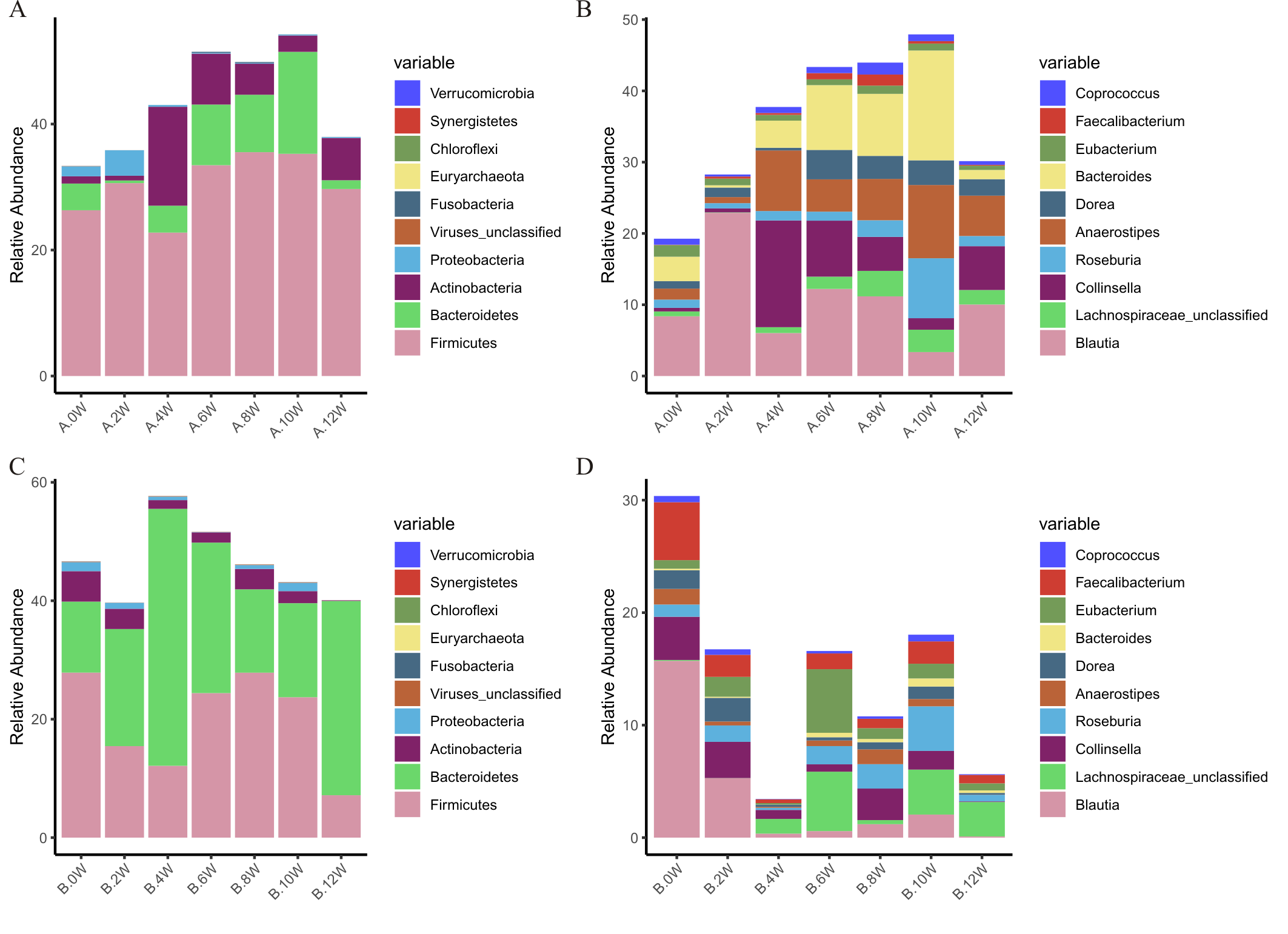

Supplement: Supplementary Figure 1 — Composition of the top 10 species at the phylum and genus level. (A). Phylum level in patient case 1. (B). Genus level in patient case 1. (C). Phylum level in patient case 2. (B). Genus level in patient case 2. [file Image_1.tif]

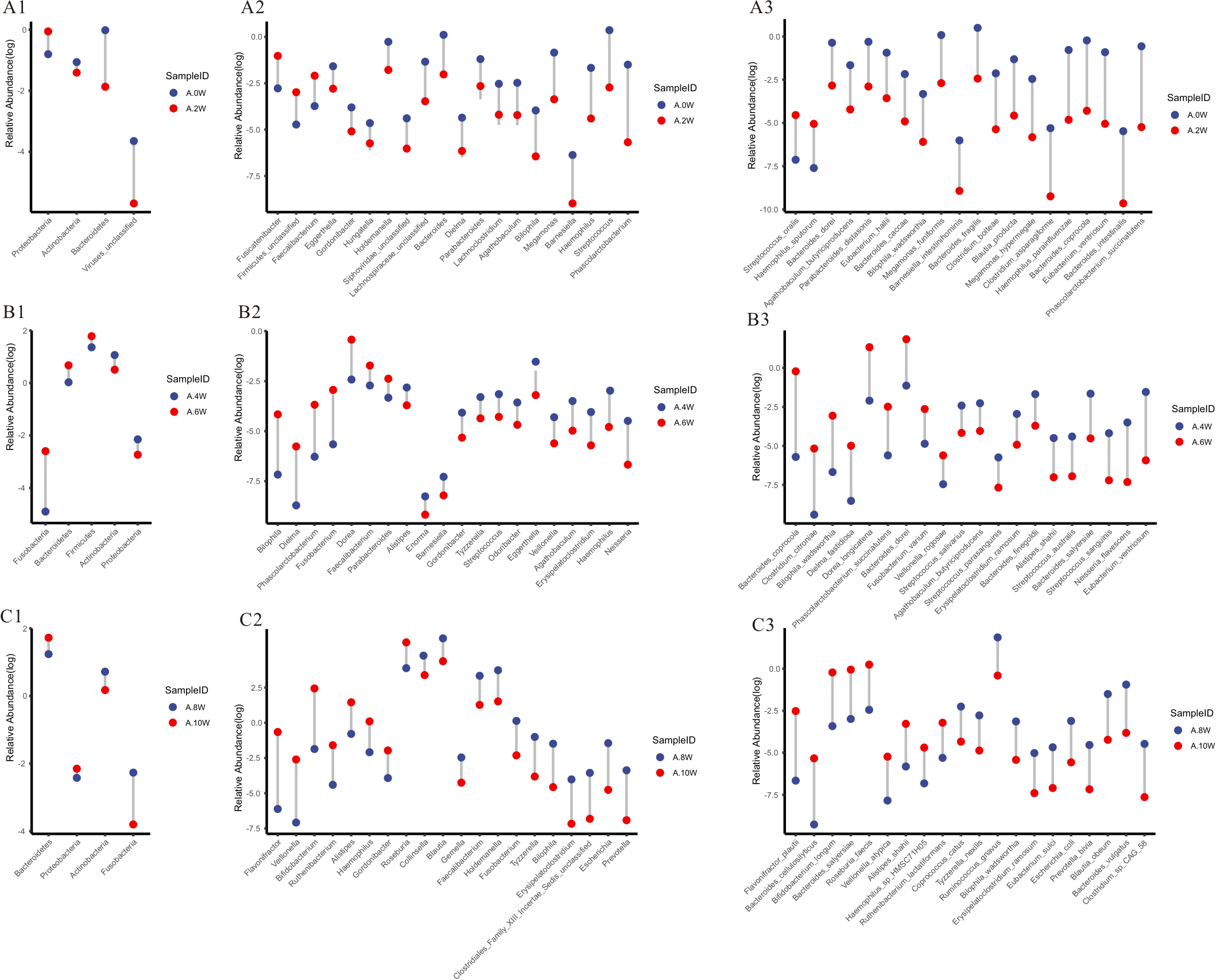

Supplement: Supplementary Figure 2 — The differences of strains at the level of phylum, and the top 20 microbiota with the most obvious differences at the level of genus and species in patient case 1. A1. The differences of strains at the level of phylum before and after use of precision probiotic strains transplantation capsule. A2. The top 20 microbiota with the most obvious differences at the level of genus before and after use of precision probiotic strains transplantation capsule. A3. The top 20 microbiota with the most obvious differences at the level of species before and after use of precision probiotic strains transplantation capsule. B1. The differences of strains at the level before and after use of fecal microbiota transplantation. B2. The top 20 microbiota with the most obvious differences at the level of genus before and after use of fecal microbiota transplantation. B3. The top 20 microbiota with the most obvious differences at the level of species before and after use of fecal microbiota transplantation. C1. The differences of strains at the level of phylum before and after use of live combined bacillus subtilis and enterococcus faecium capsules. C2. The top 20 microbiota with the most obvious differences at the level of genus before and after use of live combined bacillus subtilis and enterococcus faecium capsules. C3. The top 20 microbiota with the most obvious differences at the level of species before and after use of live combined bacillus subtilis and enterococcus faecium capsules. [file Image_2.tif]

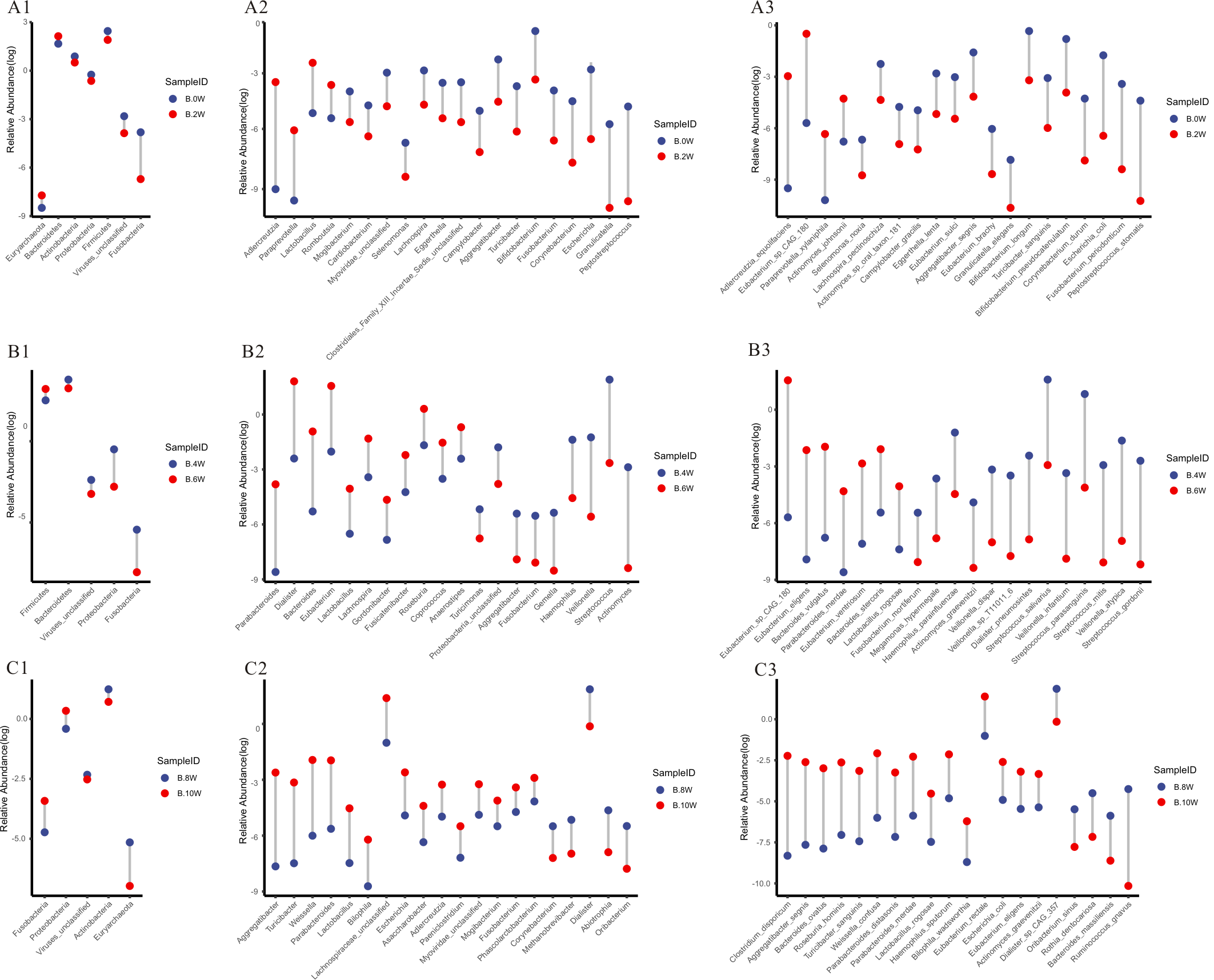

Supplement: Supplementary Figure 3 — The differences of strains at the level of phylum, and the top 20 microbiota with the most obvious differences at the level of genus and species in patient case 2. A1. The differences of strains at the level of phylum before and after use of precision probiotic strains transplantation capsule. A2. The top 20 microbiota with the most obvious differences at the level of genus before and after use of precision probiotic strains transplantation capsule. A3. The top 20 microbiota with the most obvious differences at the level of species before and after use of prcesion probiotic strains transplantation capsules. B1. The differences of strains at the level before and after use of fecal microbiota transplantation. B2. The top 20 microbiota with the most obvious differences at the level of genus before and after use of fecal microbiota transplantation. B3. The top 20 microbiota with the most obvious differences at the level of species before and after use of fecal microbiota transplantation. C1. The differences of strains at the level of phylum before and after use of live combined bacillus subtilis and enterococcus faecium capsules. C2. The top 20 microbiota with the most obvious differences at the level of genus before and after use of live combined bacillus subtilis and enterococcus faecium capsules. C3. The top 20 microbiota with the most obvious differences at the level of species before and after use of live combined bacillus subtilis and enterococcus faecium capsules. [file Image_3.tif]
