# Supplementary material for: Clinical study on sequential treatment of severe diarrhea irritable bowel syndrome with precision probiotic strains transplantation capsules, fecal microbiota transplantation capsules and live combined bacillus subtilis and enterococcus faecium capsules
Source: Front Cell Infect Microbiol. 2022 Sep 28;12:1025889. doi: 10.3389/fcimb.2022.1025889 (PMC9555570; doi:10.3389/fcimb.2022.1025889)
Supplement: Supplementary Table 1 — IBS-SSS score change. [file Table_1.docx]

Supplementary table 1.IBS-SSS score change

|  |  | **PPT** | | **FMT** | | **LBE** | |
| --- | --- | --- | --- | --- | --- | --- | --- |
|  | **Time** | **score** | **response** | **score** | **response** | **score** | **response** |
| Case1 | 0 week | 325 |  | 150 |  | 275 |  |
|  | 2 weeks | 325 | 0 | 275 | -125 | 275 | 0 |
|  | 4 weeks | 150 | **175**(R) | 275 | -125 | 300 | -25 |
| Case2 | 0 weeks | 400 |  | 340 |  | 320 |  |
|  | 2 weeks | 300 | **100**(R) | 330 | 10 | 310 | 10 |
|  | 4 weeks | 340 | **60**(R) | 320 | 20 | 320 | 0 |

IBS-SSS score decreased by at least 50 points or the severity decreased by one level, indicating clinical response. R indicate response; PPT indicate precision probiotic strains transplantation capsules. FMT indicate fecal microbiota transplantation capsules. LBE indicate live combined bacillus subtilis and enterococcus faecium capsules.

Supplementary table 2. Changes of IBS related symptoms

|  |  | **PPT** | | **FMT** | | **LBE** | |  |  |  |  |  |
| --- | --- | --- | --- | --- | --- | --- | --- | --- | --- | --- | --- | --- |
|  | **Time** | **score** | **response** | **score** | **response** | **score** | **response** |  |  |  |  |  |
| **Frequent of stool** | | | | | | | |  |  |  |  |  |
| Case1 | 0 week | 7 |  | 5 |  | 5 |  |  |  |  |  |  |
|  | 2 weeks | 4 | R | 5 |  | 4 | R |  |  |  |  |  |
|  | 4 weeks | 5 |  | 5 |  | 5 |  |  |  |  |  |  |
| Case2 | 0 week | 9 |  | 4 |  | 4 |  |  |  |  |  |  |
|  | 2 weeks | 4 | R | 5 |  | 5 |  |  |  |  |  |  |
|  | 4 weeks | 4 | R | 4 |  | 6 |  |  |  |  |  |  |
| **Character of stool** | | | | | | | |  |  |  |  |  |
| Case1 | 0 week | 7 |  | 6 |  | 6 |  |  |  |  |  |  |
|  | 2 weeks | 4 | R | 6 |  | 6 |  |  |  |  |  |  |
|  | 4 weeks | 6 |  | 6 |  | 6 |  |  |  |  |  |  |
| Case2 | 0 week | 6 |  | 4 |  | 5 |  |  |  |  |  |  |
|  | 2 weeks | 5 | R | 5 |  | 6 |  |  |  |  |  |  |
|  | 4 weeks | 4 | R | 5 |  | 6 |  |  |  |  |  |  |
| **Abdominal pain** | | | | | | | |  |  |  |  | N |
| Case1 | 0 week | 6 |  | 4 |  | 4 |  |  |  |  |  |  |
|  | 2 weeks | 4 | R | 4 |  | 4 |  |  |  |  |  |  |
|  | 4 weeks | 4 | R | 4 |  | 4 |  |  |  |  |  |  |
| Case2 | 0 week | 6 |  | 6 |  | 6 |  |  |  |  |  |  |
|  | 2 weeks | 8 |  | 6 |  | 6 |  |  |  |  |  |  |
|  | 4 weeks | 6 |  | 6 |  | 6 |  |  |  |  |  |  |
| **GSRS** | | | | | | | |  |  |  |  |  |
| Case1 | 0 week | 64 |  | 61 |  | 49 |  |  |  |  |  |  |
|  | 2 weeks | 54 | 15.6% | 53 | 13.1% | 59 | -20.4% |  |  |  |  |  |
|  | 4 weeks | 61 | 4.7% | 49 | 19.7% | 61 | -24.5% |  |  |  |  |  |
| Case2 | 0 week | 45 |  | 38 |  | 42 |  |  |  |  |  |  |
|  | 2 weeks | 43 | 4.4% | 43 | 13.2% | 38 | 9.5% |  |  |  |  |  |
|  | 4 weeks | 38 | 15.6% | 42 | 10.5% | 36 | 14.3% |  |  |  |  |  |

The times of stools more than 4 drops to 4 or less, the abdominal pain score is dropped by 30%, the GSRS score is dropped by 30%, and the stool characteristics are changed to types 3, 4, and 5 for patients which with stool types 6 and 7 were consider response. R indicate response. PPT indicate precision probiotic strains transplantation Capsules. FMT indicate fecal microbiota transplantation capsules. LBE indicate live combined bacillus subtilis and enterococcus faecium capsules.

Supplementary table 3. Changes in quality of life scale scores IBS-QoL

|  |  | **PPT** | | **FMT** | | **LBE** | |
| --- | --- | --- | --- | --- | --- | --- | --- |
|  | **Time** | **score** | **response** | **score** | **response** | **score** | **response** |
| Case1 | 0 week | 26 |  | 15 |  | 14 |  |
|  | 2 weeks | 17 | **34.6%**(R) | 15 | 0% | 10 | 28.6% |
|  | 4 weeks | 15 | **42.3%**(R) | 14 | 6.7% | 13 | 7.1% |
| Case2 | 0 weeks | 54 |  | 57 |  | 59 |  |
|  | 2 weeks | 51 | 5.6% | 61 | -7.0% | 43 | 27.1% |
|  | 4 weeks | 57 | -5.6% | 59 | -3.5% | 46 | 22.0% |

A 30% drop in IBS-QoL score indicates an improvement in quality of life. R indicate response. PPT indicate precision probiotic strains transplantation Capsules. FMT indicate fecal microbiota transplantation capsules. LBE indicate live combined bacillus subtilis and enterococcus faecium capsules.

Supplementary table 4. Changes in anxiety and depression scale scores

|  |  | **PPT** | | **FMT** | | **LBE** | |
| --- | --- | --- | --- | --- | --- | --- | --- |
|  | **Time** | **score** | **response** | **score** | **response** | **score** | **response** |
| **GAD7** |  |  |  |  |  |  |  |
| Case1 | 0 week | 5 |  | 3 |  | 2 |  |
|  | 2 weeks | 2 | R | 1 |  | 5 |  |
|  | 4 weeks | 3 | R | 2 |  | 5 |  |
| Case2 | 0 weeks | 14 |  | 10 |  | 13 |  |
|  | 2 weeks | 14 |  | 16 |  | 10 |  |
|  | 4 weeks | 10 |  | 13 |  | 10 |  |
| **PHQ9** |  |  |  |  |  |  |  |
| Case1 | 0 week | 5 |  | 6 |  | 9 |  |
|  | 2 weeks | 10 |  | 10 |  | 11 |  |
|  | 4 weeks | 6 |  | 9 |  | 11 |  |
| Case2 | 0 weeks | 14 |  | 17 |  | 16 |  |
|  | 2 weeks | 13 |  | 15 |  | 14 | R |
|  | 4 weeks | 17 |  | 16 |  | 13 | R |

The improvement of anxiety and depression is manifested in the degree of anxiety and depression drops at least one level. R indicate response. PPT indicate precision probiotic strains transplantation Capsules. FMT indicate fecal microbiota transplantation capsules. LBE indicate live combined bacillus subtilis and enterococcus faecium capsules.
